# Clinical Outcomes and Early Postoperative Complications in Boston Type I Keratoprosthesis Implantation: A Retrospective Study

**DOI:** 10.3390/jcm13226710

**Published:** 2024-11-08

**Authors:** Katarzyna Krysik, Piotr Miklaszewski, Anna Maria Gadamer, Dominika Janiszewska-Bil, Anita Lyssek-Boroń, Dariusz Dobrowolski, Beniamin Oskar Grabarek, Edward Wylęgała

**Affiliations:** 1 Department of Ophthalmology, St. Barbara Hospital, Trauma Centre, 41-200 Sosnowiec, Poland; kkrysik@gmail.com (K.K.); piotrmiklaszewski94@gmail.com (P.M.); annagadamer978@gmail.com (A.M.G.); dominika.bjaniszewska@gmail.com (D.J.-B.); anitaboron3@gmail.com (A.L.-B.); dardobmd@wp.pl (D.D.); 2 Department of Ophthalmology, Faculty of Medicine, Academy of Silesia, 40-555 Katowice, Poland; 3 Collegium Medicum, WSB University, 41-300 Dabrowa Gornicza, Poland; 4 Department of Ophthalmology, District Railway Hospital, 40-760 Katowice, Poland; wylegala@gmail.com; 5 Department of Ophthalmology, Faculty of Medicine, Medical University of Silesia, 40-555 Katowice, Poland

**Keywords:** keratoprosthesis, ocular surface disorders, dry eye, complications

## Abstract

**Background/Objectives:** The purpose of this study is to evaluate clinical outcomes and early postoperative complications in patients undergoing Boston type I keratoprosthesis (BKPro) implantation, with a specific focus on the onset and nature of ocular surface disorders during the early postoperative period. **Methods:** This retrospective study included 77 eyes that underwent BKPro implantation between 2019 and March 2022. Patients were treated at the Saint Barbara Hospital, Trauma Centre, Sosnowiec, Poland, and District Railway Hospital, Katowice, Poland. Data were collected from medical records, including patient demographics, medical history, surgical outcomes, postoperative visual acuity, and complications. The analysis incorporated both detailed medical history and direct clinical examination results. **Results:** The most common early postoperative complication was glaucoma, affecting 32 eyes (42%). Retroprosthetic membrane formation occurred in 20 eyes (26%), while partial extrusion of the BKPro was observed in 10 eyes (13%). Severe corneal surface damage was noted in patients with underlying autoimmune diseases (36%) and after chemical burns (24%). Five patients suffered from transient hypotony resulting from keratoprosthesis extrusion. The results highlight the complex nature of managing ocular surface conditions and the frequent challenges associated with early postoperative complications. **Conclusions:** BKPro implantation is an effective solution for severe corneal diseases that are resistant to conventional treatments. However, it is associated with a significant risk of early complications, particularly glaucoma and retroprosthetic membrane formation. Early identification and management of these complications are crucial for improving outcomes and maintaining visual function. Further research into optimizing postoperative care is needed to minimize these risks and enhance patient outcomes.

## 1. Introduction

Severe ocular surface diseases and anterior segment conditions often lead to significant damage, resulting in the deterioration of the cornea and surrounding tissues. These conditions may include total corneal limbal stem cell deficiency (LSCD), persistent non-healing epithelial defects, corneal melting, ulceration, superficial or deep neovascularization, and corneal scarring. In such cases, conventional treatments may fail to restore corneal integrity or visual function. The primary objectives of treatment are to achieve stable ocular surface homeostasis, maintain the structural integrity of the eyeball, and ultimately restore visual capacity [1,2].

Boston type I keratoprosthesis (BKPro) implantation is generally recommended for patients with advanced ocular surface conditions for which standard corneal transplants have failed or are unlikely to succeed [3,4]. Such cases include, but are not limited to, bilateral LSCD, multiple failed corneal grafts, severe corneal opacity with extensive neovascularization, and neurotrophic keratitis [5,6]. Additionally, BKPro is suitable for individuals with cicatrizing conjunctival diseases, including ocular cicatricial pemphigoid, Stevens–Johnson syndrome, and cases involving extensive chemical or thermal burns. The BKPro serves as an alternative treatment for patients whose conditions are so severe that other surgical interventions are considered unlikely to provide a successful outcome [7,8].

The changes over decades in the design of implants, improvement of surgical techniques, and postoperative management have substantially increased the success rate of KPro implantation and decreased its complications. Despite the careful choice of eyes for KPro implantation, postoperative complications can lead to vision loss over time. The most common complications are glaucoma, retroprosthetic membranes (RMs), aseptic keratolysis, infectious keratitis, extrusion of the KPro, endophthalmitis, hypotony, sterile uveitis, and retinal detachment [4,8,9,10,11].

Disorders in patients with BKPro are described in long-term observations, while the aim of this study is to present early complications accompanying the implantation of a BKPro and to assess their frequency. Many of the abnormalities that occur are related to the early healing process and the tissue response to the implant. Describing the changes at the initial stage of treatment may be valuable not only in choosing the optimal time to begin treatment but also in planning the appropriate pharmacological therapy.

## 2. Materials and Methods

### 2.1. Ethics

The study received approval from the Ethics Committee (No. 25/KB/AŚ/04/2024) on 3 April 2024, and was carried out in compliance with the principles outlined in the Declaration of Helsinki. All participants provided informed consent prior to their involvement in the research. Additionally, written consent was obtained from the patients for publication purposes.

### 2.2. Study Design

This study reviews the surgical treatment of 77 eyes that underwent BKPro implantation, which were operated on between 2019 and March 2022. Patients were recruited from the Ophthalmology Department of Saint Barbara Hospital, Trauma Centre, Sosnowiec, Poland, and the Chair and Clinical Department of Ophthalmology, School of Medicine with the Division of Dentistry in Zabrze, Medical University of Silesia, District Railway Hospital, Katowice, Poland. Data from the medical records included demographics, medical history, preoperative and postoperative best spectacle-corrected visual acuity (BCVA) measured using the Snellen VA chart, outcome and complications of surgery, postoperative intraocular pressure, graft rejection, and other comorbidities and complications. All patients signed an informed consent form before any surgical procedure.

### 2.3. Subjects

In order to achieve a stable ocular surface, most patients priorly underwent a frequently repetitive multistage surgery, such as keratolimbal allograft (KLAL) in 13 cases, conjunctival limbal autograft (CLAU) in 6 cases, amniotic membrane transplantation (for one-time restoration of lost conjunctival fornices or serving as a substrate for stem cell proliferation and epithelial migration, accelerating re-epithelialization of the cornea) in 21 cases, surgical treatment of adnexal pathologies, such as ectropion, entropion, trichiasis, dacryocystitis, symblepharolysis, ankyloblepharolysis, and other lid procedures, in 9 cases. The above-mentioned treatments to improve the condition of the ocular surface were necessary in the case of eyelid changes and shallowing of the eyelid.

### 2.4. BKPro Procedure

A minimum of one year after ocular surface reconstruction, BKPro was implanted. The BKPro type I consisted of three components: a front plate made of clear PMMA plastic with a central stem, a titanium back plate 8.5 mm in diameter and a titanium locking ring assembled around a donated corneal graft before insertion into the patient’s eye. The corneal graft was prepared, and a central hole was made in it with a 3 mm punch so that it would fit over the stem. The graft was then placed over the BKPro I front plate, and the assembly tool was used to push it gently down over the stem. Viscoelastic material was applied to the back surface of the graft, and the back plate was placed over the stem. The locking ring was pressed onto the stem with a finger, and the assembly tool was used to press the locking ring firmly into the groove. Then, the central patient’s opaque cornea was removed, and if the natural lens or intraocular lens was present, it was also removed. The prosthesis with the attached corneal graft was sewn with 16 10–0 nylon interrupted sutures, as in standard transplantation. 

#### Post-Operative Period

In all patients, both topical and systemic antirejection medications were administered individually and postoperatively. Intensive topical and general medical management for the primary ocular pathology was continued according to its aetiology: immunosuppression, anti-inflammatory, broad-spectrum antibiotic, antifungal, or antiprotozoal therapy, respectively. Patients were also treated with cycloplegics and antiglaucomatous medications, if necessary. All patients were hospitalized for the first 3–5 days after operation and were followed up every two weeks for two months, monthly for a minimum of six months, and at differing intervals thereafter.

### 2.5. Statistical Analysis

Statistical analysis was conducted using XLSTAT-Biomed, version 2023.1 (Addinsoft SARL, Paris, France), operating on the Windows platform. Prior to analysis, the Shapiro–Wilk test was performed to assess the normality of the data. As the data did not follow a normal distribution, they are presented as median values with the lower quartile (Q1) and upper quartile (Q3). The Wilcoxon signed-rank test, a nonparametric test appropriate for paired data, was then used to evaluate changes in visual acuity (VA) before and after Boston Keratoprosthesis surgery across different patient groups. A *p*-value of less than 0.05 was considered statistically significant for all comparisons. In this study, the chi-square test was applied to examine associations between gender and various causes leading to the implantation of BKPro.

## 3. Results

### 3.1. Patient Demographics

A total of 77 BKPro procedures were performed, including 30 in females (mean age 55.18 ± 17.4 years; range 21–85) and 47 in males (mean age 52.53 ± 15.43 years; range 24–89), with no significant age difference between genders (*p* > 0.05). Each eye underwent an average of 2.65 prior corneal transplants (range 0–13). Notably, one patient with Lyell syndrome had 13 keratoplasties, including 9 penetrating and 4 specialized grafts. The average early postoperative follow-up period was 14.2 ± 8.7 months.

### 3.2. Indications for BKPro Type I Implantation

The primary indication for BKPro was post-inflammatory corneal scarring or leucoma, affecting 45 eyes, often related to infectious keratitis with corneal inflammation, transparency loss, stromal neovascularization, and scarring. Severe autoimmune diseases were included in this subgroup. Other indications included ocular trauma in 29 eyes, primarily from alkali burns (21 eyes) and thermal burns (7 eyes). Less common cases were neurotrophic and silicon oil keratopathy, resulting in corneal edema and central scarring. A chi-square test showed no significant association between gender and either inflammation-related causes (*p* = 0.2348) or ocular trauma (*p* = 0.8011) (Table 1).

### 3.3. VA Outcomes

Table 2 shows logMAR VA outcomes before and after BKPro surgery. For infection/inflammation cases, median VA improved significantly from 0.010 (Q1: 0.010, Q3: 0.012) to 0.20 (Q1: 0.040, Q3: 0.550) (*p* < 0.00001). In autoimmune disease cases, median VA increased from 0.012 (Q1: 0.010, Q3: 0.0188) to 0.115 (Q1: 0.020, Q3: 0.275) (*p* = 0.00417). Chemical burns showed a median VA improvement from 0.010 (Q1: 0.010, Q3: 0.014) to 0.150 (Q1: 0.065, Q3: 0.300) (*p* = 0.00013). For thermal burns, median VA increased from 0.012 (Q1: 0.010, Q3: 0.016) to 0.391 (Q1: 0.0725, Q3: 0.525) (*p* = 0.01172). Overall, BKPro surgery significantly improved VA across all groups (Table 2).

### 3.4. Postoperative Complications

**Figure 1:**
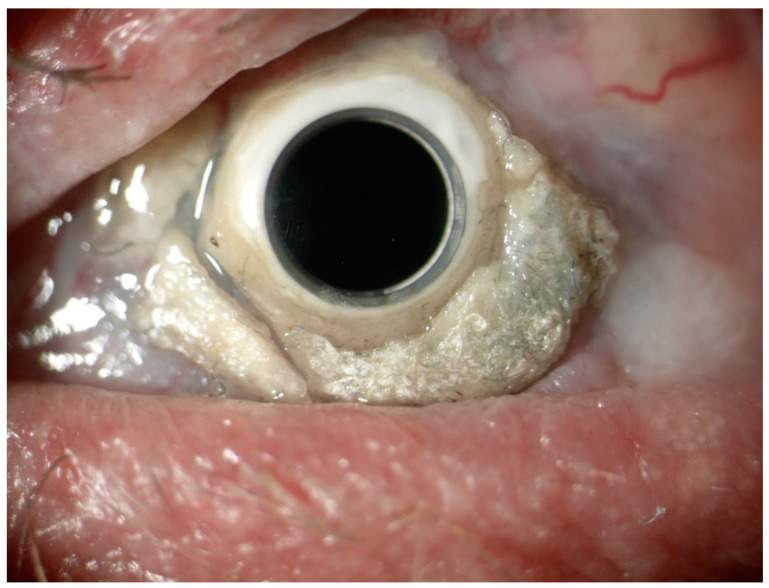
Calcifications around the implant.

**Figure 2:**
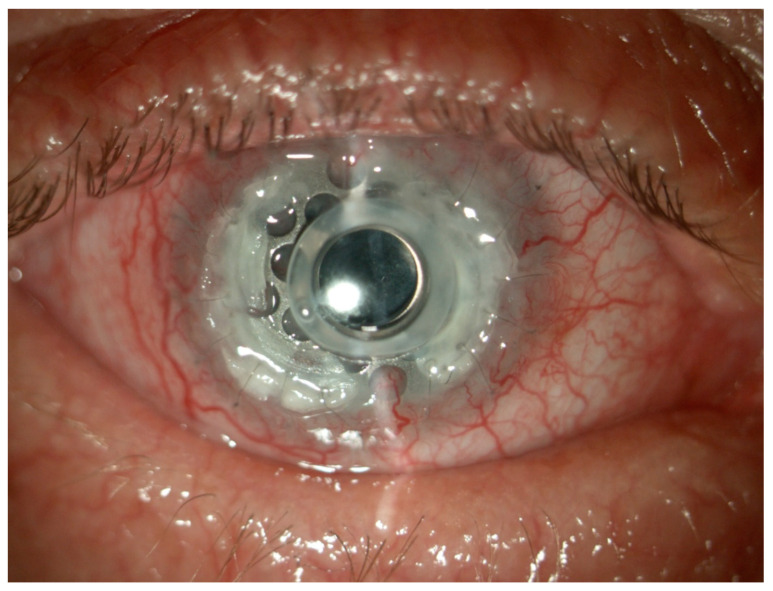
BKPro titanium ring exposure under corneal melting.

Despite careful patient selection, complications were observed postoperatively (Table 3). The most common was glaucoma or ocular hypertension, affecting 32 eyes (42%), despite peripheral iridectomy in 22 cases (29%). Other complications included retroprosthetic membrane (RM) formation in 20 eyes (26%), persistent epithelial defects in 10 eyes (13%), and aseptic keratolysis with BKPro extrusion in 7 eyes (9%). Glaucoma necessitated surgical intervention in 8 eyes (10%) via trabeculectomy or cyclophotocoagulation, with continued medication needed in some cases. RM formation led to visual deterioration, predominantly in cases of prior infectious keratitis, burns, or autoimmune disease. Aseptic keratolysis leading to BKPro extrusion was noted in seven eyes (9%) within 4 months, necessitating surgical intervention such as keratolimbal allografting. Vitreous hemorrhage, occurring in five eyes within the first 4 weeks, resolved and did not affect final VA outcomes. Figure 1 and Figure 2 illustrate complications: calcification around the implant (Figure 1) and BKPro titanium ring exposure due to corneal melting (Figure 2).

In the treatment of glaucoma among BKPro patients, 7.8% underwent laser cyclophotocoagulation, 2.6% underwent trabeculectomy, and the remainder were managed with topical therapies. Despite these interventions, visual acuity in 7.8% of patients declined to light perception. Retroprosthetic membranes were successfully treated with Nd laser membranotomy in all cases. For patients with epithelial defects, re-epithelialization was supported by the continuous application of therapeutic contact lenses and increased lubrication. Corneal melting was managed by covering the affected area with a limbal allograft, ensuring sufficient exposure of the BKPro; all interventions were successful. Patients received systemic immunosuppression with mycophenolate mofetil (500 mg BID). Table 3 summarizes the early postoperative complications observed in patients treated with BKPro.

## 4. Discussion

Corneal transplantation is one of the most frequent transplant procedures, but if it fails for the first time for any reason, a repeat transplantation using an artificial or donor cornea may be considered [7,12]. BKPro application is considered a procedure of last resort for these patients [12,13]. An appropriate candidate for a BKPro implant must be clearly informed of the potential risks of complications, including irreversible visual loss [14]. All of them are burdened with a high risk of complications. Improvement of the design and materials of the BKPro and optimization of postoperative care have considerably reduced the rate and severity of potential complications [10,12,14,15]. As Patel et al. underlined, KPro surgery, in comparison with standard penetrating keratoplasty, provides rapid visual recovery. The deterioration of vision following BKPro was due in part to progression of an underlying disease, such as glaucoma, RM formation, or retinal detachment [11].

Glaucoma after BKPro implantation is one of the most common causes of poor visual success [5,7,10,11,14,16] and loss of vision in long observations [6,15]. Glaucoma or increased intraocular pressure may be the comorbidity secondary to the underlying ocular surface pathology or frequent, previous numerous surgeries, and the use of oral and topical steroids may lead to the development of increased pressure. Better visual outcomes of BKPro implantation are seen in eyes with primary managed glaucoma. Our findings follow many others [5,6,7]. Prior glaucoma surgery, mainly glaucoma drainage device placement, has been widely advised in the literature [4,6,7,14]. In our group of patients, two eyes had previously implanted the Ahmed glaucoma valve in the operated eye, which resulted in a lack of necessity to use antiglaucomatous treatment after BKPro implantation. About 29% of our operated eyes had a one-time peripheral iridectomy performed with BKPro implantation. This form of surgery reduced the successive pharmacological treatment of increased intraocular pressure, and there was no need to operate on these eyes. Despite careful IOP measurements and both surgical and pharmacological management of pre-existing or post-BKPro implantation glaucoma, it appears to be the most significant in terms of impact on vision and treatment complication resistance. Geoffrion et al. [10] recommended reaching a low IOP target of 12 mmHg and considering administering prophylactic glaucoma medications before BKPro implantation in eyes without any preoperative diagnosis of glaucoma. Chemically burned eyes can already be compromised from structural damage to all layers of the eye, worsening the prognosis. Our report is in accordance with many others [5,10] in which damaged eyes had concomitant preoperative ocular diseases, particularly glaucoma. Severe optic nerve or retina damage due to ocular surface disease became evident only after the media were cleared from opacity.

Hou et al. [17] reported that pre-KPro KLAL, even in failed post-KLAL keratoplasty, in combination with immunosuppressive therapy, can minimize corneal melting and improve long-term retention in inflamed patients. The authors underlined that when KLAL succeeds in the long term, the patient is spared the risks of KPro implantation. Postoperative immunosuppression therapy should be tapered slowly in eyes with persistent inflammation. These findings follow our results. These findings are consistent with our results. Patients who had prior surgical reconstruction of the ocular surface using either KLAL or CLAU techniques required fewer reoperations due to BKPro extrusion or dehiscence. Sterile melts occurred in almost 50% of the eyes operated on by Iyer et al. [9], primarily in patients with chemical injuries. This complication was observed mainly in patients with autoimmune diseases (36%) and was less frequent after chemical trauma. In the majority of eyes, melting was generally an inflammatory rather than an infectious complication, which is comparable with others [13].

However, the change in BKPro type I of the PMMA backplate to titanium has decreased the rate of retro-prosthetic membrane formation [8,14] and is still one of the most frequent complications of this surgery [11,13]. The rate of RM formation in our report is comparable to the findings of previously cited authors [9]. The retro-backplate membrane is correlated with an increased risk of sterile keratolysis, possibly because of the impedance of nutritional support from the aqueous humor reported by Sivaraman et al. [18]. Optical coherence tomography evidence of an RM in their study was observed in 100% of eyes with corneal melting and in 34.1% of eyes that did not. Other studies have shown an increased risk of vitreoretinal complications, including retinal detachment (of tractional or rhegmatogenous origin), in the presence of RM [4]. However, only visually significant RM can be treated with an Nd:YAG laser. Thick and/or vascularized membranes must be excised via surgical excision and sometimes KPro explantation or exchange [8]. Greiner et al. [6] observed RM formation in 55% of operated eyes, where refractory to YAG laser membranotomy RMs (12.5%) required a surgical approach.

BKPro is an alternative treatment option for severe corneal blindness in cases of multiple unsuccessful and frequently repetitive surgical reconstructions of the ocular surface of different origins. Increased success rates are due to improvements in surgical and pharmacological treatment, as well as a better understanding of the mechanisms at the core of all complications [17,19,20,21,22]. Our results of the BKPro type I implantation show that because of the complex nature of the ocular surface pathology, it is difficult to avoid complications and determine the final result of this treatment. The differences in frequency of various surgery complications may result from selection of the study group, their medical background, preoperative diagnoses, and comorbidities. Further investigations and development of surgical techniques are necessary to improve the final results of this surgery. The main purpose should be to improve visual function with a minimum of complications.

## 5. Conclusions

Observations of patients in the early period after BKPro implantation indicate a significant percentage of disorders increasing intraocular pressure and the presence of RMs. The presence of a large implant in the anterior chamber favors both pathologies, and they should be considered together. Patients with ocular surface disorders are also at high risk of complications caused by the exposure of a portion of the BKPro above the tissue surface.

## Figures and Tables

**Table 1:** Indications for BKPro implantation.

Characteristics	Total (*n* = 77) N (%)	Female (*n* = 30) N (%)	Male (*n* = 47) N (%)	*p*-Value (Chi-Square Test)
Inflammation	45 (59.2)	23 (29.9)	22 (28.5)	0.2348
Infection/inflammation	31 (40.2)	14 (18.2)	17 (22)	
Autoimmune disease	14 (18.2)	9 (11.7)	5 (6.5)	
Ocular trauma	28 (36.8)	7 (9.1)	21 (28.6)	0.8011
Chemical burn	21 (27.3)	5 (5.25)	16 (15.8)	
Thermal burn	7 (9.1)	2 (1.75)	5 (5.2)	
Other	3 (3.9)	2 (2.6)	1 (1.3)	
Neurotrophic keratopathy	2 (2.6)	1 (1.3)	1 (1.3)	-
Silicon oil keratopathy	1 (1.3)	1 (1.3)	0	

Data are presented as number of cases (percentages).

**Table 2:** Average logMAR VA.

Characteristics	VA Before BKPro	VA After BKPro	*p*-Value
Infection/inflammation	2.00 (2.00; 1.92)	0.70 (1.40; 0.26)	<0.00001
	1.92 ± 2.42	0.48 ± 0.47	
Autoimmune disease	1.92 (2.00; 1.73)	0.94 (1.70; 0.56)	0.00417
	1.86 ± 2.33	0.74 ± 0.68	
Chemical burn	2.00 (2.00; 1.85)	0.82 (1.19; 0.52)	0.00013
	1.92 ± 2.48	0.61 ± 0.54	
Thermal burn	1.92 (2.00; 1.80)	0.41 (2.14; 0.28)	0.01172
	1.85 ± 2.35	0.37 ± 0.49	

Data are presented as median (Q1; Q3) and mean ± standard deviation.

**Table 3:** Complications observed early postoperatively.

Description	Percentage
Glaucoma/Ocular hypertension	42.1%
Retroprosthetic membrane	26.3%
Persistent epithelial defects	13.1%
Aseptic keratolysis with implant extrusion	9.2%

## Data Availability

The data used to support the findings of this study are included in the article. The data cannot be shared due to third-party rights and commercial confidentiality.
